# A case of non-bacterial thrombotic endocarditis on the aortic valve following coronary angiography

**DOI:** 10.1093/jscr/rjae212

**Published:** 2024-04-02

**Authors:** Nobuhiro Motiduki, Ryohei Ushioda, Sayaka Yuzawa, Kazuki Miyatani, Hideki Isa, Yuki Setogawa, Kohei Ishidou, Masahiko Narita, Fumitaka Suzuki, Aina Hirofuji, Ryo Okubo, Shingo Kunioka, Masahiro Tsutsui, Natsuya Ishikawa, Kamiya Hiroyuki

**Affiliations:** Department of Cardiac Surgery, Asahikawa Medical University, Midorigaoka Higashi 2-1-1-1, Asahikawa 078-8510, Japan; Department of Cardiac Surgery, Asahikawa Medical University, Midorigaoka Higashi 2-1-1-1, Asahikawa 078-8510, Japan; Department of Diagnostic Pathology, Asahikawa Medical University Hospital, Midorigaoka 1-1-1, Asahikawa 078-8510, Japan; Department of Cardiac Surgery, Asahikawa Medical University, Midorigaoka Higashi 2-1-1-1, Asahikawa 078-8510, Japan; Department of Cardiac Surgery, Asahikawa Medical University, Midorigaoka Higashi 2-1-1-1, Asahikawa 078-8510, Japan; Department of Cardiac Surgery, Asahikawa Medical University, Midorigaoka Higashi 2-1-1-1, Asahikawa 078-8510, Japan; Department of Cardiac Surgery, Asahikawa Medical University, Midorigaoka Higashi 2-1-1-1, Asahikawa 078-8510, Japan; Department of Cardiac Surgery, Asahikawa Medical University, Midorigaoka Higashi 2-1-1-1, Asahikawa 078-8510, Japan; Department of Cardiac Surgery, Asahikawa Medical University, Midorigaoka Higashi 2-1-1-1, Asahikawa 078-8510, Japan; Department of Cardiac Surgery, Asahikawa Medical University, Midorigaoka Higashi 2-1-1-1, Asahikawa 078-8510, Japan; Department of Cardiac Surgery, Asahikawa Medical University, Midorigaoka Higashi 2-1-1-1, Asahikawa 078-8510, Japan; Department of Cardiac Surgery, Asahikawa Medical University, Midorigaoka Higashi 2-1-1-1, Asahikawa 078-8510, Japan; Department of Cardiac Surgery, Asahikawa Medical University, Midorigaoka Higashi 2-1-1-1, Asahikawa 078-8510, Japan; Department of Cardiac Surgery, Asahikawa Medical University, Midorigaoka Higashi 2-1-1-1, Asahikawa 078-8510, Japan; Department of Cardiac Surgery, Asahikawa Medical University, Midorigaoka Higashi 2-1-1-1, Asahikawa 078-8510, Japan

**Keywords:** nonbacterial thrombotic endocarditis, acute cerebral infarcts

## Abstract

Nonbacterial thrombotic endocarditis (NBTE) on the aortic valve involves fibrin and platelet aggregate formation, potentially leading to embolic events. We present a case of NBTE on the aortic valve following coronary angiography (CAG) in a 54-year-old man with multiple comorbidities. Surgical thrombectomy was performed owing to acute cerebral infarcts. This case highlights the significance of considering that mechanical trauma from catheterization during CAG can trigger thrombus formation.

## Introduction

Nonbacterial thrombotic endocarditis (NBTE) is a clinical syndrome characterized by valvular thickening or vegetations composed of fibrin and platelet aggregates without apparent signs of inflammation or bacterial involvement [1]. It frequently causes cerebral infarction and other embolisms. Reject thrombosis or anticoagulation therapy is the primary treatment for NBTE [2, 3]. However, in contrast to infectious endocarditis, the surgical indications and postoperative course remain unclear. The general etiology and pathogenesis of NBTE involve endothelial damage, leading to subendothelial exposure to circulating platelets [4]. Mechanical factors including localized blood turbulence or those induced by catheters can lead to endothelial damage [4, 5]. We here report the case of surgical thrombectomy for NBTE on the aortic valve occurring following coronary angiography (CAG).

## Case report

**Figure 1:**
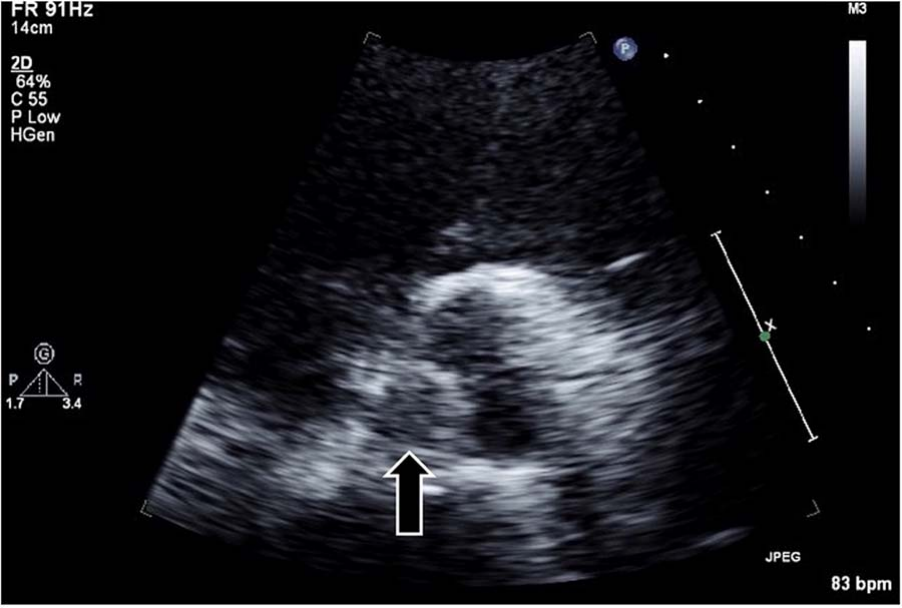
Transthoracic echocardiography in the parasternal short-axis view revealing a hypo- or iso-echoic mass (arrow; ~1 cm in diameter) on the noncoronary cusp of the aortic valve.

A 54-year-old man presented to our emergency department with shortness of breath on exertion. He had a medical history of hypertension, diabetes, and gastric ulcer. Transthoracic echocardiography (TTE) revealed a low left ventricular ejection fraction (30%). CAG revealed normal left coronary; however, the catheter could not access the abnormally positioned right side. No aortic valve abnormalities or vegetation were observed in TTE or CAG. Seven days post-CAG, TTE revealed a 1-cm vegetation and edematous changes at the noncoronary cusp of the aortic valve (Fig. 1). Laboratory data showed that the cause of thrombus formation was not identified (anticardiolipin IgG, 12.4 APL U/ml; anticardiolipin IgM, 2.9 APL U/ml; protein C, 52%; and protein S, 43%). Brain magnetic resonance imaging revealed multiple acute cerebral infarct lesions. Therefore, the patient underwent urgent thrombectomy.

**Figure 2:**
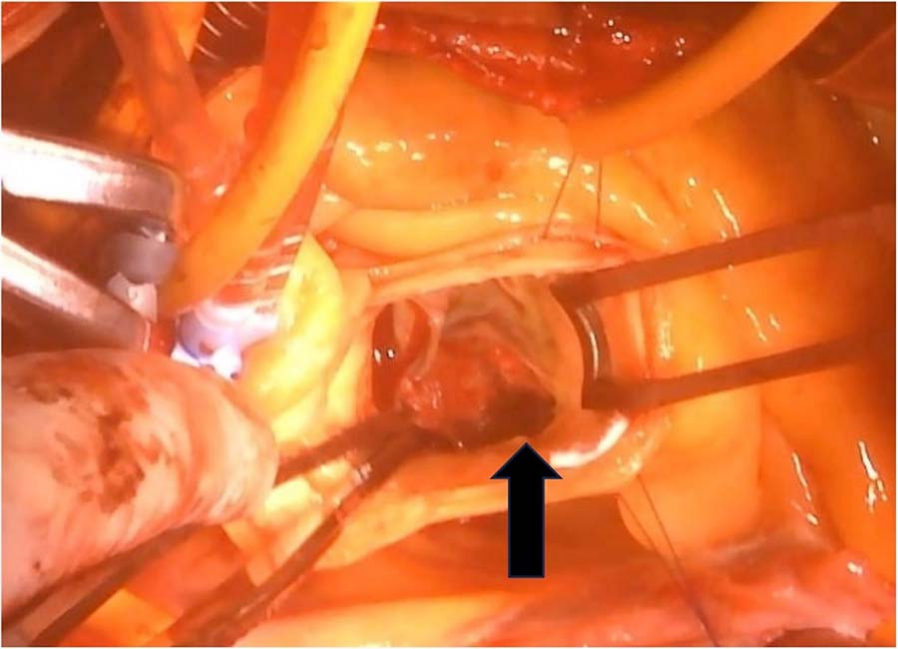
A 2 × 2-cm vegetation is observed on the noncoronary cusp of the aortic valve.

**Figure 3:**
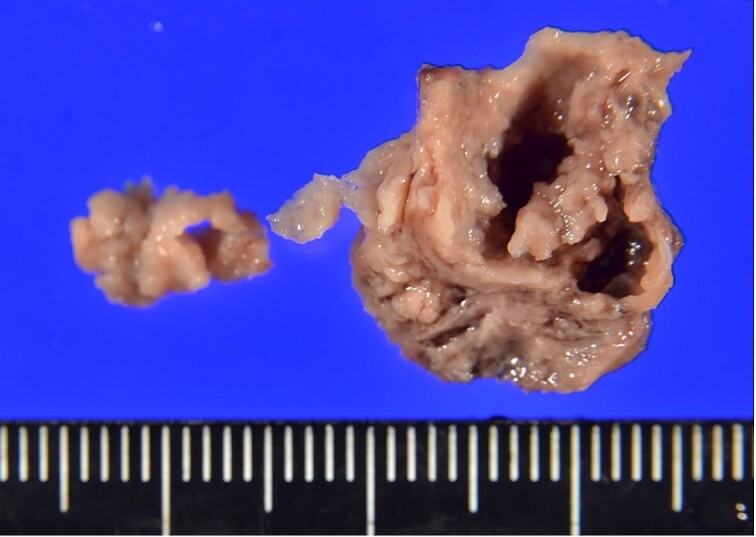
Gross appearance of the resected vegetation.

**Figure 4:**
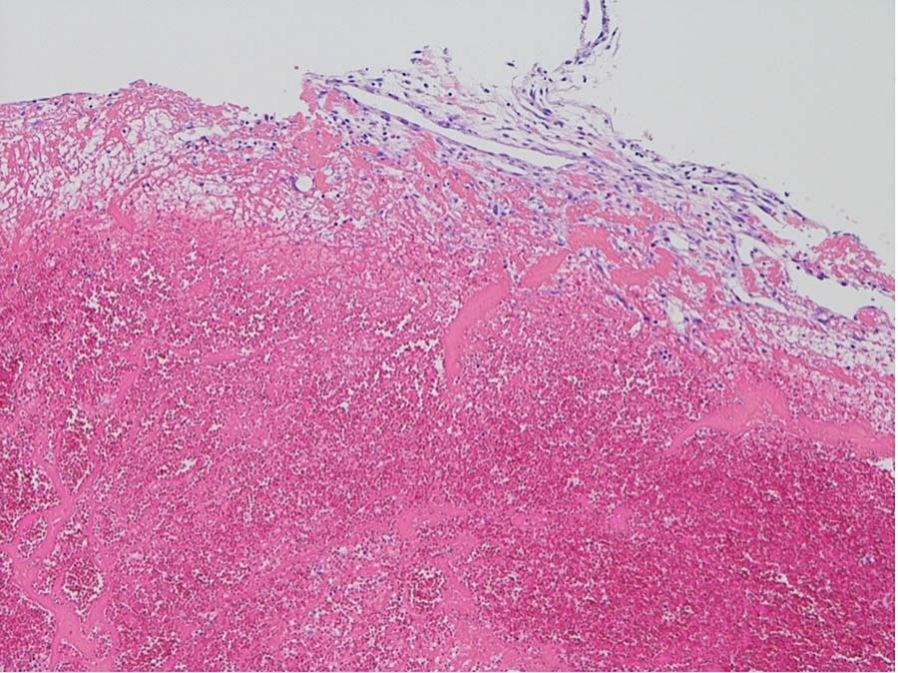
Histopathological appearance of the vegetation: mainly fresh and organizing thrombus, consisting of fibroblast and capillary hyperplasia in the upper part of the specimen (hematoxylin and eosin staining, ×100).

The surgical procedure employed a median sternotomy, with creation of cardiopulmonary bypass via cannulation of the ascending aorta and right atrium. After cross-clamping, cold antegrade cardioplegia was administered. Upon aortic valve exposure, a 2 × 2-cm vegetation was noted on the noncoronary cusp (Figs 2 and 3). The vegetation was quickly removed, and the noncoronary cusp was preserved. Following a 50-min bypass time, with a 35-min cross-clamp time, the patient was successfully weaned off bypass with minimal inotropic support. A portion of the vegetation was sent for culture; however, no bacterial or fungal growth was detected. Further histopathologic examination revealed a fibrin-rich thrombus containing red blood cells with organization at the periphery (Fig. 4).

Postoperatively, the patient experienced atrial fibrillation and consequently developed a mobile thrombus at the left atrial appendage despite sufficient anticoagulation. On postoperative Day 14, the patient underwent left appendage closure and pulmonary vein isolation using a radiofrequency energy device (AtriCure, Mason, OH, USA). On postoperative Day 30 following the initial surgery, the patient was discharged. One year following discharge, the patient remains well without any thromboembolic events.

## Discussion

In 1888, Zeigler [4] first proposed NBTE as a valvar thrombus, distinguishing it from infectious endocarditis. Approximately all NBTE cases are incidentally detected in the anatomic pathology department. Jara *et al.* [6] noted 22 cases (1.08%) of NBTE in 2041 adult autopsies. NBTE primarily affects the mitral valves, with rare cases of aortic valve involvement [2]. Infarction events are the main complication of NBTE. When NBTE is detected on the aortic valve, we mainly must pay attention to myocardial infarction because the coronary artery is directly above it [1, 7]. Our patient had asymptomatic multiple cerebral infarctions without myocardial infarction.

NBTE frequently involves a systemic hypercoagulable state, including malignancy, systemic lupus erythematosus, antiphospholipid syndrome, polycythemia, and proteins C and S deficiencies [1–4]. The precise trigger for thrombus formation remains unclear. However, immune complex or mechanical endothelial injury sets in motion a cascade leading to platelet aggregate and fibrin strand deposition on cardiac structures [4, 5, 8]. Histologically, a fresh and organized thrombus, characterized by fibroblast and capillary hyperplasia, is observed within the vegetation adhering to the valve surface. Our case was diagnosed as NBTE on the basis of the abovementioned histological features; however, the cause of thrombus formation was not identified. Conversely, of note, vegetation developed in the aortic valve following CAG. As the right coronary artery originated from the left coronary apex, catheterizing the right coronary artery was difficult. Attempting multiple cannulations may have damaged the endocardium of the aortic valve. A previous case report showed aortic valve vegetation following radiofrequency catheter ablation of a left-sided accessory pathway via a retrograde approach [9]. Mechanical stimulation by the catheter may have induced thrombus formation.

Anticoagulation therapy to dissolve the thrombus and prevent recurrent thromboembolism is the primary treatment of NBTE [1–4, 6, 7]. Although some cases have shown resolution of valvular thrombus with anticoagulation, surgical intervention becomes necessary when valvular dysfunction leads to uncontrolled heart failure or when recurrent thromboembolism persists despite anticoagulation [2–4, 9–11]. Alajaji *et al.* [1] suggested that thrombectomy in native aortic valves can yield better outcomes than isolated antithrombotic management. However, as underlying diseases contributing to NBTE can increase operative mortality, surgical intervention should be approached cautiously [2]. We performed the surgery to protect against additional embolism due multiple cerebral infarct formation. Regardless of postoperative anticoagulation, thrombus recurrence occurred, possibly exacerbated by postoperative atrial fibrillation. Several studies have reported thrombus recurrence in patients with NBTE [10, 11]. Therefore, promptly initiating postoperative anticoagulation upon achieving hemostasis, even before a definitive diagnosis, is advisable. Considering the potential for short-period recurrences, we recommend a short-term follow-up, as shown in this case.

In conclusion, we report a unique case of NBTE on the aortic valve following CAG. Mechanical stimulation from the catheter may have triggered thrombus formation. Despite postoperative anticoagulation administration, thrombus recurrence was observed. Therefore, patients with NBTE who undergo thrombectomy should have short-term follow-up for monitoring.

## References

[1] Alajaji W , Hornick JM, Malek E, et al. The characteristics and outcomes of native aortic valve thrombosis: a systematic review. J Am Coll Cardiol 2021;78:811–24. 10.1016/j.jacc.2021.06.023. 34412815

[2] Quintero-Martinez JA , Hindy JR, El Zein S, et al. Contemporary demographics, diagnostics and outcomes in non-bacterial thrombotic endocarditis. Heart 2022;108:1637–43. 10.1136/heartjnl-2022-320970. 35534050

[3] Zmaili MA , Alzubi JM, Kocyigit D, et al. A contemporary 20-year Cleveland clinic experience of nonbacterial thrombotic endocarditis: etiology, echocardiographic imaging, management, and outcomes. Am J Med 2021;134:361–9. 10.1016/j.amjmed.2020.06.047. 32827467

[4] Asopa S , Patel A, Khan OA, et al. Non-bacterial thrombotic endocarditis. Eur J Cardiothorac Surg 2007;32:696–701. 10.1016/j.ejcts.2007.07.029. 17881239

[5] Saito M , Asano N, Ota K, et al. Three mitral valve operations in a patient with trousseau syndrome and nonbacterial thrombotic endocarditis caused by ovarian cancer. Kyobu Geka 2016;69:1067–71. 27909274

[6] Llenas-García J , Guerra-Vales JM, Montes-Moreno S, et al. Nonbacterial thrombotic endocarditis: clinicopathologic study of a necropsy series. Rev Esp Cardiol 2007;60:493–500. 10.1016/S0300-8932(07)75066-2. 17535760

[7] Eguchi K , Ohtaki E, Misu K, et al. Acute myocardial infarction caused by embolism of thrombus in the right coronary sinus of Valsalva: a case report and review of the literature. J Am Soc Echocardiogr 2004;17:173–7. 10.1016/j.echo.2003.10.002. 14752493

[8] Zmaili M , Alzubi J, Lo Presti Vega S, et al. Non-bacterial thrombotic endocarditis: a state-of-the-art contemporary review. Prog Cardiovasc Dis 2022;74:99–110. 10.1016/j.pcad.2022.10.009. 36279942

[9] Raitt MH , Schwaegler B, Pearlman AS, et al. Development of an aortic valve mass after radiofrequency catheter ablation. Pacing Clin Electrophysiol 1993;16:2064–72. 10.1111/j.1540-8159.1993.tb01002.x. 7694255

[10] Yuriditsky E , Torres J, Izmirly PM, et al. Resolution of large aortic valve vegetations in antiphospholipid syndrome treated with therapeutic anticoagulation: a report of two cases and literature review. Lupus 2018;27:2269–73. 10.1177/0961203318804876. 30290716

[11] Svantner J , Lavanchy L, Labouchère A. Recurrent embolic strokes due to antiphospholipid syndrome and non-bacterial thrombotic endocarditis in a patient with basal cell carcinoma. J Cardiothorac Surg 2023;18:177. 10.1186/s13019-023-02266-6. 37170311 PMC10176810

